# Infant and young child feeding practices among children under 2 years of age and maternal exposure to infant and young child feeding messages and promotions in Dar es Salaam, Tanzania

**DOI:** 10.1111/mcn.12292

**Published:** 2016-04-15

**Authors:** Bineti S. Vitta, Margaret Benjamin, Alissa M. Pries, Mary Champeny, Elizabeth Zehner, Sandra L. Huffman

**Affiliations:** ^1^ Consultant to Helen Keller International; ^2^ Helen Keller International Dar es Salaam Tanzania; ^3^ Helen Keller International Asia Pacific Regional Office Phnom Penh Cambodia; ^4^ Helen Keller International 1120 20th St, NW Suite 500 N Washington, DC 20036 USA

## Abstract

There are limited data describing infant and young child feeding practices (IYCF) in urban Tanzania. This study assessed the types of foods consumed by children under 2 years of age and maternal exposure to promotions of these foods in Dar es Salaam, Tanzania. A cross‐sectional survey was conducted among 305 mothers of children less than 24 months of age who attended child health services in October and November, 2014. Among infants less than 6 months of age, rates of exclusive breastfeeding were low (40.8%) and a high proportion (38.2%) received semi‐solid foods. Continued breastfeeding among 20–23‐month‐olds was only 33.3%. Consumption of breastmilk substitutes was not prevalent, and only 3.9% of infants less than 6 months of age and 4.8% of 6–23 month‐olds were fed formula. Among 6–23‐month‐olds, only 38.4% consumed a minimum acceptable diet (using a modified definition). The homemade complementary foods consumed by the majority of 6‐23‐month‐olds (85.2%) were cereal‐dominated and infrequently contained micronutrient‐rich ingredients. Only 3.1% of 6–23‐month‐olds consumed commercially produced infant cereal on the day preceding the interview. In contrast, commercially produced snack foods were consumed by 23.1% of 6–23‐month‐olds. Maternal exposure to commercial promotions of breastmilk substitutes and commercially produced complementary foods was low (10.5% and 1.0%, respectively), while exposure to promotions of commercially produced snack foods was high (45.9%). Strategies are needed to improve IYCF practices, particularly with regard to exclusive and continued breastfeeding, increased dietary diversity and consumption of micronutrient‐rich foods, and avoidance of feeding commercially produced snack foods.

## Introduction

Chronic malnutrition is highly prevalent in Tanzania at an early age, and over a third (35.5%) of children under 2 years of age are stunted [calculated from 2010 Tanzania Demographic and Health Survey (TDHS) data; National Bureau of Statistics (NBS) & ICF Macro 2011]. Micronutrient deficiencies among children are common, and the prevalences of iron deficiency, anaemia and vitamin A deficiency among 6–23‐month‐olds are 42%, 73% and 33%, respectively (calculated from TDHS data; NBS & ICF Macro 2011; *Micronutrients*). Sub‐optimal infant and young child feeding (IYCF) practices are major contributors to childhood malnutrition. Results from the 2010 TDHS show low rates of exclusive breastfeeding (49.8%; defined as breastfeeding and consuming no water, non‐milk liquids/juice, other milk or complementary foods in the 24 h prior to the interview among 0–5‐month‐olds), continued breastfeeding at 2 years of age (49.0%) and feeding in accordance with recommended IYCF practices (21.3%), an outcome similar to the World Health Organization (WHO) minimum acceptable diet indicator (NBS & ICF Macro 2011). An analysis of TDHS data by Huffman *et al.* showed that, among children 6–23 months of age, sugary foods are more commonly consumed than micronutrient‐rich foods such as fortified infant cereals, eggs and vitamin‐A rich fruits (13% vs. 4%, 8% and 0%, respectively; 2014).

Infant and young child feeding practices can be influenced by maternal exposure to promotions and messaging (Piwoz & Huffman 2015; Vaga *et al.* 2014). In response to unethical marketing activities by breastmilk substitute companies, the WHO developed the 1981 *International Code of Marketing of Breast‐milk Substitutes* (WHO 1981). In 1994, Tanzania adopted the *Code* by instituting *The National Regulations for Marketing of Breast‐milk Substitutes and Designated Products* (Government of Tanzania 1994). This legislation was updated in 2013 to become the *Tanzania Food, Drugs and Cosmetics (Marketing of Foods and Designated Products for Infants and Young Children),* which prohibited the promotion of infant formula, follow‐up formula, growing‐up milks and ‘any product marketed, or otherwise represented or commonly used for feeding of infants…or beverages, milks and other foods intended for use by infants and young children’ (Government of Tanzania 2013). This legislation covers products marketed for children up to 5 years of age in its definition of ‘young children’, thereby restricting promotion of any product marketed for children under 5 years of age. There are no Tanzanian laws that restrict the marketing of commercially produced snack foods to the general public. Understanding the IYCF messages mothers in Tanzania receive from the health system is needed in order to reinforce positive messages and encourage consumption of appropriate complementary foods, while understanding the IYCF promotions mothers are exposed to from the manufacturers and distributors of commercially produced IYCF products is needed in order to discourage possible inappropriate promotion of commercially produced foods for infants and young children (including breastmilk substitutes and commercially produced snack foods).

**Key messages**

- Several infant and young child feeding indicators are suboptimal in Dar es Salaam, Tanzania.
- Rates of breast‐milk substitute and commercially produced complementary food consumption are low, while the consumption of commercially produced snack food products is common.
- Maternal exposure to infant and young child feeding messaging and commercial promotions of commercially produced infant and young child foods is low, but common for commercially produced snack food products.
- Strategies are needed to increase rates of exclusive breastfeeding, continued breastfeeding to two years, and minimum dietary diversity, and decrease commercially produced snack food consumption.

Aside from the TDHS data, there are limited data describing IYCF practices and dietary intakes of children in Tanzania and maternal exposure to IYCF messaging and commercial promotions, particularly in Dar es Salaam, its largest city. These data are needed to better understand the scope and nature of suboptimal IYCF practices and factors affecting them in order to inform the development of appropriate interventions. The objectives of this study were to measure (1) IYCF indicators; (2) rates of consumption of homemade and commercially produced infant and young child foods as well as commercially produced snack foods; and (3) maternal exposure to IYCF messaging and promotions of commercially produced infant and young child foods and snack foods. This study was part of a larger four‐country study to examine promotional practices of foods fed to infants and young children as well as their consumption in the largest metropolitan areas of Cambodia, Nepal, Senegal and Tanzania.

## Materials and methods

### Study design and study population

This was a cross‐sectional survey using a multi‐stage sampling procedure to obtain a representative sample of mothers of children <24 months of age attending urban health centers in Dar es Salaam. Data were collected through structured interviews. The study population was composed of mothers of children aged 0–23 months, currently living in Dar es Salaam, who were visiting health services with their child between 6 October and 19 November 2014. Sampling mothers at health facilities served as a proxy for sampling mothers from the general population. Prior research has indicated that commercially produced infant and young child foods are more widely available in urban areas than rural areas (Sweet *et al.* 2012); therefore, it was concluded that Dar es Salaam, the largest city in Tanzania, would be an appropriate site to assess promotion and consumption of these products. Mothers of children with congenital diseases, mothers of children who had been in the neonatal intensive care unit (NICU), mothers who had experienced severe delivery complications or mothers of children from a multiple birth were excluded from the study because of these characteristics potentially impeding successful breastfeeding. Mothers with a child too ill for interview were also excluded.

### Sample size

The sample size for this study was calculated to estimate a 10% prevalence of consumption of commercially produced infant and young child foods among children less than 2 years of age on the day preceding the interview and a 10% prevalence of maternal exposure to promotions of such products, to within 5 percentage points. A prevalence of 10% was selected as this was viewed as a level that warranted concern. Using a standard error of 0.025 and assuming a design effect of 2 to account for the cluster design, a sample size of 280 mothers of children less than 24 months of age was considered adequate. Because of the cluster sampling design utilized (described later), the final sample size was slightly higher than 280, and a total of 305 mothers were interviewed.

### Sampling procedure

Lists of all health facilities offering child health services in Dar es Salaam were obtained from the Tanzania Ministry of Health Database. Health facilities owned by the government and non‐governmental or faith‐based organizations were included, while facilities that were privately owned were excluded. Health posts were excluded because of the small number of attendees. Private facilities were excluded because utilization of private facilities in Tanzania is relatively low (6.0% of live births in Dar es Salaam occurred in private health facilities in 2010; NBS & ICF Macro 2011). The types of health facilities that remained in the sampling frame consisted of referral hospitals, health centres and dispensaries.

**Figure 1:**
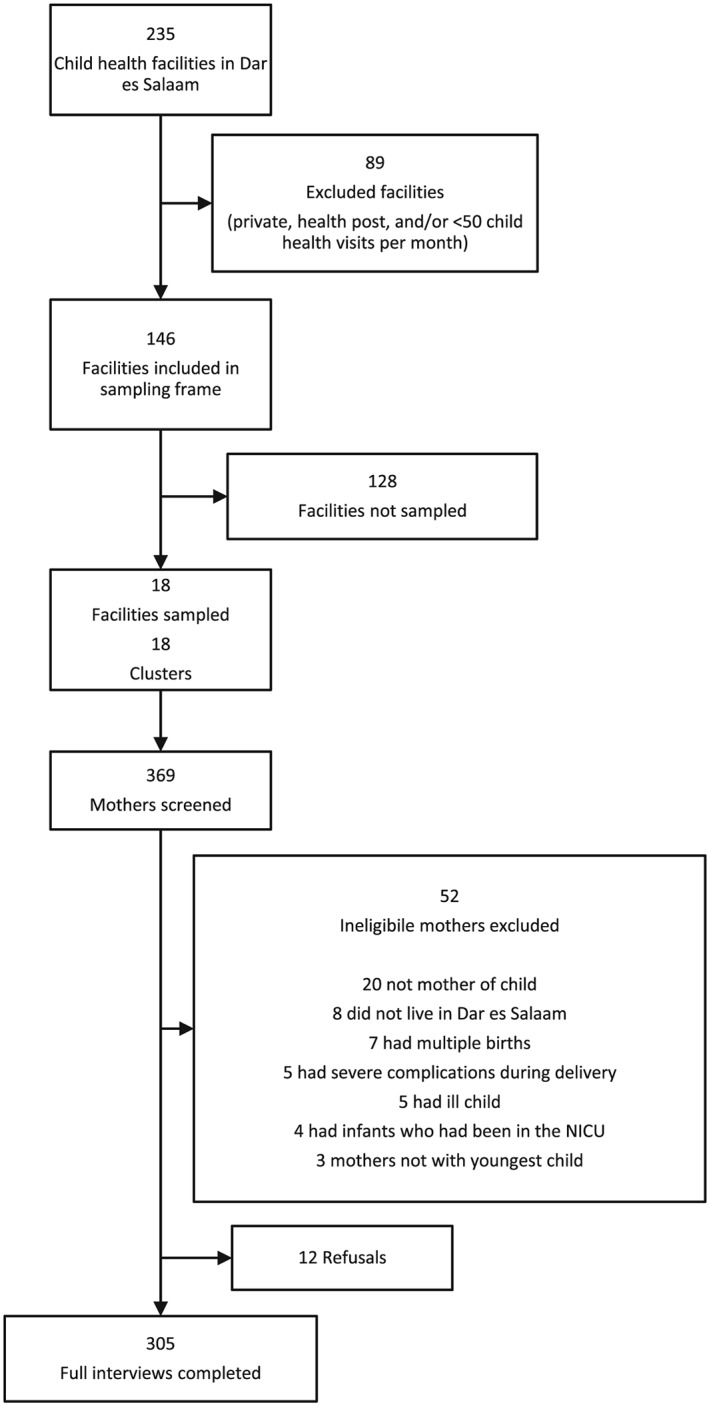
Health facility sampling and participant flow.

In order to complete data collection within 8–10 weeks, facilities with less than 50 child health visits per month were excluded from the sampling frame. Average monthly utilization numbers for each health facility were determined using information from the Tanzania Ministry of Health Database on the total number of child health visits during the period of August 2013–June 2014. The exclusion of privately owned health facilities, health posts, and facilities with less than 50 child health visits per month resulted in the exclusion of 89 out of 235 child health facilities (Fig. 1). Based on the facility utilization numbers, the 146 child health facilities included in the sampling frame is where 76% of all child health visits took place in Dar es Salaam from August 2013–June 2014.

Health facilities were then sampled by allocating clusters using probability proportional to size. The calculated average monthly utilization numbers served as each facility's ‘population’. Clusters (*n* = 18) of 16 mothers each were assigned across facilities in the sampling frame. Stratified sampling was implemented to enroll an equal number of children (*n* = 4) across all four age categories (0–5, 6–11, 12–17 and 18–23 months). Eighteen clusters from 18 health facilities were included in the study. Of the 369 mothers approached for interview, 12 mothers refused to be interviewed, and 52 were excluded due to ineligibility (i.e. 20 were not the mother of the child, eight did not live in Dar es Salaam, seven had a child from a multiple birth, five had severe complications during delivery, five were with a child too ill for interview, four had a child who had been in the NICU and three were not with their youngest child). Completed interviews were obtained for 305 mothers (Fig. 1).

### Data collection

Sampled facilities were provided with information on the dates when data collection would take place, approximately 1 week prior to survey and again 1–2 days before the study team's arrival. Study supervisors screened women with children in the child health clinic and paediatric outpatient department areas to assess if (1) they were the mother of the child present; (2) their child was under 24 months of age; and (3) they lived in Dar es Salaam. Supervisors also asked the mother for the child's date of birth to calculate the age of the child to determine which age stratum the child belonged to and whether the sample size requirements for that stratum at that clinic had been achieved. The exclusion criteria questions asked by the supervisors during screening were also asked again by enumerators in the formal questionnaire used for interviewing mothers.

Ethical clearance for this study was obtained from Muhimbili University of Health and Allied Sciences, Directorate of Research and Publications (Ref No. MU/DRP/AEC/Vol.XVIII/130, 31 July 2014) and the Tanzanian Commission for Science and Technology prior to data collection. Permission to collect data at the selected facilities was obtained from district level authorities. Verbal informed consent was obtained from all participants prior to the interview.

Information was collected on maternal demographic and socio‐economic characteristics (including age, parity, marital status and educational attainment), receipt of antenatal care and delivery assistance, household assets, drinking water source and child characteristics (including age, gender and if delivered by caesarean section). Data on IYCF practices were gathered in accordance with the WHO guidelines on IYCF practices (WHO 2008). Standardized questionnaires were used to obtain information on the foods and liquids consumed by children on the day and night prior to the interview. Additionally, data were gathered on reasons for feeding the child certain foods and the amount of money spent on commercially produced snack food purchases for the child. Mothers were also asked to recall their exposure to IYCF messaging, promotions of commercially produced infant and young child foods and promotions of commercially produced snack foods.

Data were collected using Ona, an open‐source online platform that allows data to be collected via phones or tablets (Ona 2014), using the Android application Open Data Kit Collect (OpenDataKit 2014). Data were submitted online to a web‐based database. The questionnaires were translated from English into Swahili, back translated into English to ensure accuracy and uploaded into Ona in Swahili. Interviews were conducted in Swahili using the Samsung Galaxy Tab 2 and 3 model tablets. Submitted questionnaires were reviewed weekly to ensure data quality.

Twelve women were trained as enumerators over a 5‐day period. Training included a pre/post training evaluation of enumerators' understanding of the information presented in training, a detailed review of the questionnaire and instruction on how to use the tablets and software. Enumerators obtained additional training and practice during pretesting, which took place at two health facilities not selected for inclusion in the study that were purposively selected for pretesting based on location and estimated patient flow. During pretesting, supervisors observed interviews and evaluated enumerators. Feedback was provided to enumerators directly after observation, at the end of each day of pretesting and after data had been uploaded to Ona and checked for any problems needing review.

### Statistical analyses

Data were cleaned and analyzed using spss version 21 (IBM Corp., Armonk, NY). IYCF indicators were calculated according to WHO definitions (WHO 2008). Univariate results were presented as proportions and means ± standard deviation (SD). Bivariate associations were assessed using 2‐sided Pearson's chi‐square tests and 2‐sided Fisher's exact tests.

## Results

### Demographic and socio‐economic characteristics

Demographic and socio‐economic characteristics for mothers and children are shown in Table 1. On average, mothers were 26.5 years of age. Almost half (44.4%) of the mothers were primiparas. About 94% had attended any level of formal education, with 2.3% having attended university. Twenty‐three percent of mothers reported working outside of the home, and 92.5% reported themselves to be the main caregiver of their youngest child. The majority (86.6%) of mothers received antenatal care during their pregnancy from a health professional or a community health worker. Close to half (48.2%) received a breastfeeding message during antenatal care, with 38.7%, 13.1% and 5.9% having received a message on exclusive breastfeeding to 6 months, continued breastfeeding to 2 years and early initiation of breastfeeding, respectively. Almost all (96.7%) mothers had delivered their youngest child with the assistance of a health professional, and 12.5% had delivered their child via caesarean section. The mean age of children was 11.8 months, as would be expected given the effort made to sample children across an equal distribution of ages 0–23 months. Three‐fourths (76.4%) of mothers reported having a safe source of drinking water for their household. A high proportion of mothers reported that their household owned a mobile phone (90.5%), had electricity (64.3%) or owned a television (57.7%); however, ownership of a refrigerator was less common (33.1%).

**Table 1:** Demographic and socio‐economic characteristics of mothers and children (*n* = 305)

**Maternal characteristics**	
Age (years) (mean ± SD)*	26.5 ± 5.8
Parity (number) (mean ± SD)**	2.0 ± 1.3
Marital status (%)	
Married or living with a man	86.6
Separated, divorced, or widowed	3.6
Never married and never lived with a man	9.8
Level of education (%)†	
None	5.9
Pre‐primary	0.3
Primary	61.6
Lower secondary	21.6
Upper secondary	6.9
Tertiary education	2.3
Works outside the home (%)	22.6
Main caregiver of the child (%)	92.5
Received antenatal care from health professional or community health worker (%)	86.6
Delivery assisted by health professional (%)	96.7
**Child characteristics**	
Age (months)(mean ± SD)	11.8 ± 6.6
Sex (female) (%)	51.8
Caesarean section delivery (%)	12.5
**Household characteristics**	
Safe source of drinking water (%)	76.4
Assets, ownership (%)	
Mobile phone	90.5
Electricity	64.3
Television	57.7
Refrigerator	33.1

* *n* = 303; 2 mothers (0.7% of total sample) missing data on age.

** *n* = 304; 1 mother (0.3% of total sample) missing data on parity.

† *n* = 301; 4 mothers (1.3% of total sample) missing data on level of education.

### Infant and young child feeding practices

#### Breastfeeding and bottle feeding practices and breastmilk substitute use

Breastfeeding and bottle feeding rates are shown in Table 2. All but one mother (99.7%) had ever breastfed their child. Breastfeeding rates decreased with age (*P* < 0.001): all children less than 6 months of age, almost all (96.2%) 6–11‐month‐olds and a high proportion (84.7%) of 12–17‐month‐olds were breastfed during the day prior to the interview. Less than half (46.2%) of 18–23‐month‐olds were reported to have been breastfed on the previous day. Among infants less than 6 months of age, 40.8% were exclusively breastfed and 53.9% were predominantly breastfed. Close to half (42.1%) received plain water, and over a third (38.2%) were fed soft, semi‐solid or solid foods on the previous day. The provision of water increased with age: 8.3% (1/12) of 0–1‐month‐olds consumed water on the previous day compared with 31.4% (11/35) of 2–3‐month‐olds and 69% (20/29) of 4–5‐month‐olds (*P* < 0.001). Soft, semi‐solid or solid food consumption was more common among 3–5‐month‐olds than 0–2‐month‐olds (48.9% vs. 20.7%, *P* = 0.014). Breastmilk substitute use was low with only 3.9% of children less than 6 months of age and 4.8% of 6–23‐month‐olds having consumed a breastmilk substitute on the day preceding the interview. Among all children, 12.5% had been fed from a bottle the previous day. The most common liquids bottle fed to children were fresh cow's milk, plain water, juice and infant formula, which were bottle fed to 2.6%, 2.3%, 2.0% and 2.0% of all children, respectively.

**Table 2:** Breastfeeding and bottle feeding practices among mothers of children <24 months of age on the day preceding the interview

	*n*	%
Ever breastfed	305	99.7
Currently breastfeeding		
0–5 months	76	100.0
6–11 months	78	96.2
12–17 months	72	84.7
18–23 months	78	46.2
Exclusive breastfeeding*, **	76	40.8
Predominant breastfeeding*, †	76	53.9
Continued breastfeeding at 1 year‡	53	83.0
Continued breastfeeding at 2 years¶	42	33.3
Bottle feeding		
0–5 months	76	10.5
6–11 months	79	21.5
12–17 months	72	11.1
18–23 months	78	6.4

* Among children 0–5 months of age.

** Defined as an infant receiving breastmilk in the 24 h preceding the interview, with the allowance of oral rehydration salts, or drops or syrups (vitamins, minerals and medicines), but nothing else (WHO 2008).

† Defined as an infant receiving breastmilk in the 24 h preceding the interview, with the allowance of certain liquids, including water and water‐based drinks, fruit juice, ritual liquids, oral rehydration salts and drops or syrups (vitamins, minerals and medicines), but no other foods or liquids (in particular, non‐human milk or food‐based fluids) (WHO 2008).

‡ Among children 12–15 months of age.

¶ Among children 20–23 months of age.

### Complementary feeding practices

Complementary feeding indicators are presented in Table 3. Minimum dietary diversity and minimum meal frequency were met by 49.3% and 70.3% of children 6–23 months of age, respectively. WHO defines a minimum acceptable diet as a diet that meets the requirements of both prior indicators with the additional constraint that non‐breastfed children must receive at least two milk feedings on the previous day (2008). Because data were not collected on the number of milk feedings consumed by non‐breastfed children (24.5% of 6–23‐month‐olds in the study) on the previous day, a modified definition of minimum acceptable diet was used that did not include this criterion but included whether any milk product was consumed. Thus, the modified version of a minimum acceptable diet was consumed by just over a third (38.4%) of children 6–23 months of age.

**Table 3:** Proportion of children 6–23 months of age that met complementary feeding indicators (*n* = 229)

Minimum dietary diversity (%)*	49.3
Minimum meal frequency (%)**	70.3
Minimum acceptable diet (%)†	38.4

* Defined as the consumption of foods from at least four out of seven food groups on the previous day (WHO 2008).

** Defined as the consumption of solid, semi‐solid or soft foods ≥2 times for breastfed children 6–8 months of age, ≥3 times for breastfed children 9–23 months of age and ≥4 times for non‐breastfed children 6–23 months of age on the previous day (WHO 2008).

† Defined as the consumption of a diet that meets both minimum dietary diversity and minimum meal frequency requirements (modified version of the WHO indicator definition [2008] and does not include the criterion of non‐breastfed children consuming ≥2 milk feedings on the previous day).

Regarding consumption of specific micronutrient‐rich foods, on the previous day, 52.0% consumed flesh foods (i.e. meat, poultry, fish, seafood and organ meats), 37.6% consumed dark green leafy vegetables, 33.6% consumed yellow/orange vitamin A‐rich vegetables (e.g. pumpkin, carrots and yellow/orange sweet potatoes), 8.3% consumed vitamin A‐rich fruits and 7.4% consumed eggs. Children were fed these micronutrient‐rich foods in homemade complementary foods made specifically for the child and not consumed by other members of the family as well as in foods made for and consumed by the general family that were also fed to the child. Younger children were less likely to be fed micronutrient‐rich foods than older children. Vitamin A‐rich fruits and vegetables (i.e. vitamin A‐rich fruits, yellow/orange vitamin A‐rich vegetables and dark green leafy vegetables) were consumed by 43.0% (34/79) of 6–11‐month‐olds vs. 68.0% (102/150) of 12–23‐month‐olds (*P* < 0.001), and flesh foods were consumed by 29.1% (23/79) of 6–11‐month‐olds vs. 64% (96/150) of 12–23‐month‐olds (*P* < 0.001). Eggs were equally rarely consumed between the two age groups [6–11 months: 4.0% (3/75) vs. 12–23 months: 9.6% (14/146), *P* = 0.140].

### Consumption of homemade and commercially produced complementary foods

Homemade complementary foods made specifically for the child and not consumed by other family members were consumed by 85.2% of 6–23‐month‐old children on the day prior to interview and by 91.7% in the week prior to the interview. Two‐thirds (66.8%) of mothers reported feeding a homemade complementary food to their child every day in the week prior to the interview. The proportion of children that consumed homemade complementary foods on the day prior to the interview did not differ by age (6–11 months: 90.7%, 12–17 months: 92.6%, 18–23 months: 82.1%, *P* = 0.101). Porridge was the most commonly consumed home‐prepared complementary food, consumed by over three‐quarters (78.2%) of 6–23‐month‐olds, followed by cooking bananas (plantains), which were consumed by 19.7%.

The proportions of 6–23‐month‐olds who consumed homemade complementary foods that contained specific ingredients on the day preceding the interview were as follows: cereals (80.8%), sugar/honey (58.5%), fats/oils (43.7%) and starchy foods (i.e. potatoes, cassava, taro, white sweet potato and cooking bananas; 31.0%). With regard to micronutrient‐rich ingredients, 27.7%, 14.8%, 12.7%, 7.0%, 3.1%, 2.6% and 0.4% of 6–23‐month‐olds consumed a homemade complementary food on the previous day containing nuts, yellow/orange vitamin A‐rich vegetables, flesh foods, dark green leafy vegetables, eggs, beans or lentils and vitamin A‐rich fruit, respectively. Homemade complementary foods containing flesh foods were more frequently consumed by older children [6–11‐months: 7.4% (5/68) vs. 12–23 months: 18.9% (24/127), *P* = 0.031]. The rates of consumption of homemade complementary foods containing vitamin A‐rich fruits or vegetables and the rates of consumption of homemade complementary foods containing eggs were similar between 6–11‐month‐olds and 12–23‐month‐olds [16.5% (13/79) vs. 19.3% (29/150), *P* = 0.593; and 1.5% (1/68) vs. 4.7% (6/127), *P* = 0.425, respectively].

Of the mothers who fed their child a homemade complementary food on the day prior to interview, 41.5% reported that they fed this food because it was ‘healthy’, 21.0% of these mothers reported feeding this food because ‘the child liked it’ and 20.0% reported feeding this food because it was ‘traditionally fed’. Rates of feeding the child homemade complementary foods were similar between mothers who worked and mothers who did not [92.6% (63/68) vs. 86.3% (132/153), *P* = 0.175], mothers who were married and those who were not [88.9% (169/190) vs. 83.9% (26/31), *P* = 0.379], mothers who had attended at least secondary school and those who had not [89.6% (60/67) vs. 89.4% (132/151), *P* = 0.654] and, using refrigerator ownership as a proxy for wealth, between mothers who owned a refrigerator and those who did not [89.0% (65/73) vs. 87.8% (130/148), *P* = 0.794].

Consumption of commercially produced infant cereal was less common than consumption of homemade complementary foods, with only 3.1% of 6–23‐month‐olds having consumed a commercially produced infant cereal on the day preceding the interview.

### Consumption of commercially produced snack foods

**Figure 2:**
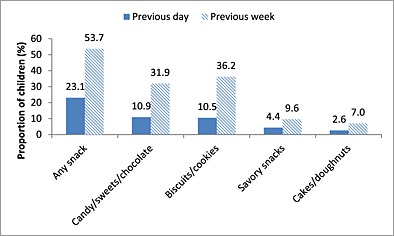
Proportion of 6–23‐month‐olds who consumed various commercially produced snack foods on the day and in the week prior to the interview (*n* = 229).

Consumption of commercially produced snack foods for general consumption (defined as sugary snack foods such as candy/sweets/chocolate, biscuits/cookies, cakes/doughnuts; and savoury snack foods such as chips, crisps or savoury biscuits marketed to the general population) on the day and in the week prior to the interview among children 6–23 months of age is shown in Fig. 2. Overall, about one quarter (23.1%) of all 6–23‐month‐old children had consumed any commercially produced snack foods on the day prior to the interview, and over half (53.7%) had consumed one in the week prior to the interview. One‐fifth (20.5%) of 6–23‐month‐olds consumed commercially produced sugary snack foods on the day prior to the interview. Candy/sweets/chocolate were consumed by 10.9% of 6–23‐month‐olds on the previous day, biscuits/cookies by 10.5% and cakes/doughnuts by 2.6%. In the previous week, candy/sweets/chocolate were consumed by 31.9% of 6–23‐month‐olds, biscuits/cookies by 36.2% and cakes/doughnuts by 7.0%. Savoury snack foods were consumed by 4.4% and 9.6% of 6–23‐month‐olds on the day and week prior to the interview, respectively. Rates of commercially produced snack food consumption differed significantly with age: 8.0% of children 6–11 months of age, 33.8% of children 12–17 months of age and 39.7% of children 18–23 months of age consumed a commercially produced snack foods on the day prior to the interview (*P* < 0.001).

Among the mothers who fed their child a commercially produced snack food in the week prior to the interview, the majority reported that their main reason for feeding the snack food to their child was because ‘the child liked it’. This reason was given by 68.7% (67/83) of mothers who fed their child biscuits/cookies, 86.3% (63/73) of mothers who fed their child candy/sweets/chocolate, 68.2% (15/22) of mothers who fed their child savoury snack foods and 87.5% (14/16) of mothers who fed their child cakes/doughnuts. Mothers who reported purchasing commercially produced snack foods in the week prior to the interview reported spending (mean ± SD) USD 0.09 ± 0.13 per day on savoury snack, foods USD 0.08 ± 0.09 per day on cakes/doughnuts, USD 0.04 ± 0.04 per day on cookies/biscuits and USD 0.02 ± 0.03 per day on candy/chocolate/sweets [1 USD = 1983.75 Tanzanian shillings (XE 2015)].

## Maternal exposure to infant and young child feeding messaging and commercially produced food product promotions

### Maternal exposure to infant and young child feeding messaging and counselling

Slightly over half (55.7%) of mothers reported hearing, seeing or receiving an educational message on IYCF since their child was born. Health facilities were the most commonly reported source of information: 42.6% of mothers reported receiving IYCF educational messages from health facilities, while 9.5% reported receiving IYCF educational messages from relatives, 8.9% from friends or neighbours, 4.3% from radio and 3.3% from television. Half (50.5%) of all mothers reported receiving exclusive breastfeeding messages, and 16.7% reported receiving continued breastfeeding messages from a health worker during antenatal care or from a health facility since their youngest child was born with no statistically significant differences reported by age of the child (data not shown). The receipt of complementary feeding messages from a health facility was less common: 9.8% of mothers reported receiving a message on introducing soft, semi‐solid or solid foods at 6 months of age, and 9.8% reported receiving a message on dietary diversity since their youngest child was born.

The proportion of mothers that reported receiving recommendations from a health professional (since the birth of their youngest child) to use breastmilk substitutes was relatively low (9.2%). Mothers who received a recommendation from a health professional to use a breastmilk substitute were more likely to do so on the day prior to the interview than mothers who did not receive a recommendation [14.3% (4/28) vs. 3.6% (10/277), *P* = 0.030]. Mothers who had delivered by caesarean section were more likely to have received a recommendation from a health professional to use a breastmilk substitute than mothers who had vaginal deliveries [34.2% (13/38) vs. 5.6% (15/267), *P* < 0.001]. The proportions of mothers of 6–23‐month‐olds that reported receiving a recommendation from a health worker to feed their child a specific food were as follows: 62.0% for fruit, 59.0% for vegetables, 20.1% for butter or oil, 18.3% for meat, 15.3% for commercially produced complementary foods and 11.4% for commercially processed cereals or grains. Very few mothers received recommendations to feed their child supplements (8.3% for micronutrient powders and 0.4% for lipid nutrient supplements).

### Maternal exposure to breastmilk substitute and commercially produced complementary food promotions

Observations since birth of the youngest child of commercial promotions for a breastmilk substitute (in a health facility, store, pharmacy or magazine; or on a billboard, the radio or television) were reported by 10.5% of mothers with no statistically significant differences seen by age of the child (data not shown). Only 1.0% reported observing a commercial promotion for a commercially produced complementary food (which they reported to be either in a magazine or on television). No mothers reported receiving free samples of breastmilk substitutes or commercially produced complementary foods since the birth of their youngest child. Two mothers reported receiving discounts/coupons on breastmilk substitutes, but no mothers reported receiving discounts/coupons on commercially produced complementary foods.

### Maternal exposure to commercially produced snack foods promotions

**Figure 3:**
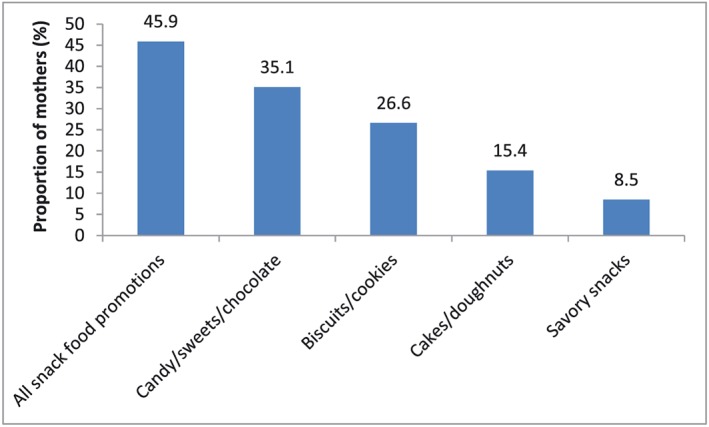
Proportion of mothers of children <24 months who were exposed to commercial promotions of commercially produced snack foods since the birth of their youngest child (*n* = 305).

Exposure to promotions of commercially produced snack foods since the birth of the youngest child was much more commonly reported by mothers than for breastmilk substitutes or commercially produced complementary foods, and close to half of all mothers (45.9%) reported observing such a promotion. The proportion of mothers who observed promotions of each type of commercially produced snack food ranged from 8.5% to 35.1% (Fig. 3). Among 6–23‐month‐olds, rates of consumption of commercially produced snack foods on the previous day were similar between children of mothers who were and were not exposed to promotions of commercially produced snack foods [24.8% (28/113) vs. 21.6% (25/116), *P* = 0.563].

## Discussion

The results of this study indicate that in Dar es Salaam, the rates of several key IYCF indicators (including exclusive breastfeeding, continued breastfeeding at 2 years and minimum dietary diversity) and maternal exposure to IYCF messaging are suboptimal. The rates of consumption and promotion of breastmilk substitutes and commercially produced complementary foods are low, while there are substantially higher rates of consumption and promotion of commercially produced snack foods. The very low rates of maternal exposure to promotions of breastmilk substitutes and commercially produced complementary foods and the low rates of use of these products are likely related to Tanzania's strict regulations limiting their promotion [*Tanzania Food, Drugs and Cosmetics (Marketing of foods and designated products for infants and young children) Regulations, 2013;* Government of Tanzania 2013]. In Senegal, where the promotion of breastmilk substitutes is allowed outside of the health system, breastmilk substitute use is more prevalent: about 10% of infants less than 6 months of age and 20% of 6–23‐month‐olds in Dakar received a breastmilk substitute on the day preceding the interview compared with 4% and 5%, respectively, in Dar es Salaam (Feeley *et al.* In press).

In the TDHS, among women aged 15–49 years residing in Dar es Salaam, rates of education, antenatal care, assisted delivery and caesarean section delivery were similar to those of the study sample suggesting that the study sample was representative of the city. In the TDHS, 6.9% of women had not received any level of formal education (vs. 5.9% in the study), 100.0% had received antenatal care from a skilled provider (vs. 86.6% in the study), 91.0% had delivered with the assistance of a health professional (vs. 96.7% in the study) and 13.4% had delivered via caesarean section (vs. 12.5% in the study; NBS & ICF Macro 2011). These were the only demographic and health variables that were directly comparable between the study and the TDHS.

The TDHS does not present IYCF outcomes among urban children alone so the study rates are compared with those of the entire TDHS sample, which is predominantly comprised of rural women and children. The IYCF rates from this study and those in or derived from the TDHS are comparable, although, they differ in a manner that seems consistent with the solely urban makeup of the study sample vs. the majority rural composition of the TDHS sample. Compared with the TDHS, this study showed lower exclusive breastfeeding rates (40.8% vs. 49.8%), higher rates of formula use among children less than 6 months of age (3.9% vs. 1.0%), lower rates of continued breastfeeding at 2 years (33.3% vs. 51.0%) and a higher prevalence of children with complementary food diets meeting minimum dietary diversity (49.3% vs. 38.2%; NBS & ICF Macro 2011; Victor *et al.* 2014). These differences could potentially be explained by reasons such as urban mothers tending to be less likely to exclusively breastfeed and continue to breastfeed for shorter durations than rural mothers (Victor *et al.* 2013), be more likely to use breastmilk substitutes, and have higher incomes that allow food variety to be more affordable. The study findings pertaining to the consumption of commercially produced snack foods are similar to those from secondary analyses of TDHS data regarding urban 6–23‐month‐olds (Huffman *et al.* 2014). Sugary snack foods were consumed by about a fifth of children (20.5% in this study vs. 23% in the secondary analyses), and the consumption of commercially produced snack foods was more common than that of most micronutrient‐rich foods (Huffman *et al.* 2014).

The study data suggest that the early cessation of exclusive breastfeeding in Dar es Salaam is largely attributable to the early provision of plain water and foods to infants, while breastmilk substitute use plays a much smaller role. In this study, while less than 4% of children under 6 months received a breastmilk substitute, 42% received water, and 38% received semi‐solid or solid foods. The frequent provision of water and foods to infants suggests the need for strategies to promote maternal understanding and acceptance of the nutritional adequacy of exclusive breastfeeding to 6 months of age and discourage the early introduction of water or foods into the child's diet. Only 9.8% of mothers reported hearing messages to delay the introduction of complementary foods to 6 months of age; thus, more information on this practice from health workers may be useful.

The results of this study also show that complementary food diets are dominated by homemade complementary foods made from cereals or grains to which sweeteners such as sugar and honey are often added (58.5% of homemade complementary foods contained sugar or honey), and micronutrient‐rich ingredients are uncommonly included (only 18.3% contained vitamin A‐rich fruit or vegetables, 12.7% flesh foods and 3.1% eggs). Thus, these homemade complementary foods are likely to be micronutrient‐poor. Micronutrient‐rich foods were often less frequently consumed among 6–11‐month‐olds than 12–23‐month‐olds. Given that 6–23‐month‐olds consume relatively small amounts of complementary foods, the nutrient density (i.e. amount of each nutrient per 100 kcal of food) of these foods needs to be very high, particularly in the second 6 months of life when nutrient density needs are highest for key problem nutrients such as iron and zinc (Pan American Health Organization & WHO 2003; Dewey 2013). Low dietary diversity has been associated with poor micronutrient density (Moursi *et al.* 2008) and child nutritional status among 6–23‐month‐olds (Arimond & Ruel 2004); thus, efforts that encourage the feeding of diverse and micronutrient‐rich foods and discourage their late introduction into the child's diet are essential for addressing child malnutrition. If it is not feasible to meet the micronutrient needs of children using locally available foods, other strategies for consideration include the use of commercially produced fortified infant cereals, when affordable, and point‐of‐use fortificants such as micronutrient powders (Feed the Future, 2015).

The results of this study suggest that the consumption of commercially produced snack foods is common, which is concerning for a number of reasons. Firstly, these calorically dense nutrient‐poor foods can contribute to childhood overweight and obesity, a public health problem which is already rapidly increasing worldwide, particularly in lower‐middle income countries and urban settings (WHO 2015). Thus, the 6% prevalence of overweight/obesity among urban Tanzanian children in 2010 is likely to increase alongside the consumption of these high calorie snack foods (NBS & ICF Macro 2011). Secondly, commercially produced snack foods have high sugar or salt contents, which are associated with the development of non‐communicable diseases including diabetes and cardiovascular disease (WHO & Food and Agriculture Organization 2003). Thirdly, the consumption of commercially produced snack foods can displace the consumption of nutrient‐rich foods by causing the child to feel satiated from calories, leaving less room for the consumption of healthful foods, and their purchase can result in less money being available for the purchase of more nutrient‐rich foods. In this study, the average amount of money spent on snack foods per day ranged from 0.02–0.09 USD, a substantial amount considering that, per 100 kcal serving, a locally produced fortified complementary food costs 0.05 USD and an imported one costs 0.69 USD in Tanzania (Pereira *et al.* 2014). Regulations restricting the promotion of commercially produced snack foods could help reduce their rates of consumption.

The low rates of maternal exposure to IYCF messaging in this study indicate the need for stronger strategies to increase the reach of IYCF messaging, especially in health facilities, mothers' primary source of IYCF messages. The results imply that messages pertaining to continued breastfeeding and complementary feeding require greater emphasis, and messages pertaining to the avoidance of feeding children commercially produced snack foods would be an important addition. Health workers should be equipped with up‐to‐date information on optimal IYCF practices to ensure the provision of appropriate messages to mothers, and a greater investment should be made in their training and support. Increasing maternal exposure to IYCF messaging may also require implementing other means of outreach including media (e.g. TV and radio commercials, billboards, additional posters in health facilities, cell phone messaging, etc.), which would need to be accompanied with safeguards to prevent commercial exploitation, and the use of outreach activities in other sectors (e.g. agriculture, bed net distribution, microcredit, WASH). More than 90% of mothers have cell phones making this an important possibility for reaching mothers over other commercial media.

The limitations of this study include those associated with the use of self‐report data. Sources of response bias include mothers potentially misreporting events that occurred due to not remembering them accurately or wanting to under‐report undesirable behaviours and over‐report desirable behaviours to appear more favourable to the interviewer. These biases would lead to the rates presented in this study differing from the true population rates. Another study limitation is the sole inclusion of mothers attending health facilities for child health services. Mothers who do not attend health facilities would have less exposure to the IYCF messaging provided there, and they may also differ from mothers who attend health facilities in other ways that would lead to differences in how they feed their child and the promotions that they are exposed to. Thus, the results presented in this study may not be generalizable to all urban mothers, but more specific to urban mothers who attend health facilities for child health services. Because the sample size of the study was based on the consumption rates of commercially produced foods, the accuracy of the prevalence estimates of other outcomes in the study are less certain as they may have required a greater sample size to generate an estimate at a similar level of precision.

A better understanding of the reasons behind suboptimal IYCF practices in Tanzania, including what motivates mothers to implement the practices that they do, is needed in order to generate effective context‐specific interventions. Such interventions will likely need to include behaviour change communication strategies to change caregivers' knowledge and attitudes, efforts to make nutrient‐rich foods more available and affordable, as well as strategies to strengthen systems that deliver IYCF counselling services and support to mothers.

## Source of funding

Bill & Melinda Gates Foundation

## Conflicts of interest statement

The authors have no conflicts of interest to declare.

## Contributor statement

BSV was involved in data collection, analysis, interpretation and writing of the paper. SLH was involved in the study design. MB was involved in the study design and data collection. AMP was involved in the study design and data collection and analysis. MC was involved in the data collection tool development. EZ was involved in the study design. All authors were involved in the review and editing of the manuscript drafts and decision to submit the paper for publication.
